# Effect of internal cleavage site mutations in human immunodeficiency virus type 1 capsid protein on its structure and function

**DOI:** 10.1002/2211-5463.12094

**Published:** 2016-06-30

**Authors:** Ferenc Tóth, János Kádas, János András Mótyán, József Tőzsér

**Affiliations:** ^1^ Department of Biochemistry and Molecular BiologyFaculty of MedicineUniversity of DebrecenHungary

**Keywords:** capsid protein, circular dichroism spectroscopy, cyclophilin A, HIV‐1, human immunodeficiency virus type 1, protease, mutagenesis

## Abstract

The capsid protein of the human immunodeficiency virus type 1 has been found to be a substrate of the retroviral protease *in vitro*, and its processing was predicted to be strongly dependent on a pH‐induced conformational change. Several protease cleavage sites have been identified within the capsid protein, but the importance of its cleavage by the viral protease at the early phase of infection is controversial. To confirm the relevance of this process, we aimed to design, produce, and characterize mutant capsid proteins, in which the protein susceptibility toward HIV‐1 protease is altered without affecting other steps of the viral life cycle. Our results indicate that while the introduced mutations changed the cleavage rate at the mutated sites of the capsid protein by HIV‐1 protease, some of them caused only negligible or moderate structural changes (A78V, L189F, and L189I). However, the effects of other mutations (W23A, A77P, and L189P) were dramatic, as assessed by secondary structure determination or cyclophilin A‐binding assay. Based on our observations, the L189F mutant capsid remains structurally and functionally unchanged and may therefore be the best candidate for use in studies aimed at better understanding the role of the protease in the early postentry events of viral infection or retrovirus‐mediated gene transduction.

**Abbreviations**
CA: capsid protein
CypA: Cyclophilin A
PR: protease

HIV‐1 is a member of the retrovirus family and is a causative agent of acquired immunodeficiency syndrome. The advance in highly active antiretroviral therapy (HAART) had dramatically changed the outcome of disease progression; however, a definitive cure is not yet available. The total number of people living with HIV infection is estimated to be over 36 million worldwide, according to the 2014 report of UNAIDS on the global AIDS epidemic. On the other hand, HIV‐based Vesicular stomatitis virus‐pseudotyped self‐inactivating vectors are among the most frequently utilized in gene therapy trials.

In mature virions, approximately 1500 capsid (CA) monomers form a conical structure—called the core—which contains the viral genome, some viral (nucleocapsid, reverse transcriptase, integrase, Vpr, and p6) and cellular (cyclophilin A, APOBEC3G) proteins, that introduce the viral genome into target cells 1. Earlier results strongly suggested that appropriate stability of the core also had a main role in HIV‐1 infection 2, 3, 4, 5, 6, 7 influencing the decapsidation process in the early phase where the exact identity of the factors involved is still not fully resolved. Besides the presence of various viral proteins, the protease (PR) is also part of the core 8 and, therefore, it might contribute to the viral infectivity by further processing viral proteins or by cleaving cellular ones 9. At the late phase of the viral life cycle, proteolytic cleavage of Gag and Gag‐Pol polyproteins is essential for the production of the mature, infectious virus 10. *In vitro* experiment with the cores of another lentivirus; the equine infectious anemia virus, showed that the viral protease can cleave the nucleocapsid protein into smaller fragments 11 leading to the suggestion of an early phase role for the PR. In previous studies it was established that proteins of the viral core (CA, NC) are substrates of the viral protease *in vitro* 12, 13, 14, while other studies identified over 30 cellular proteins that could be target of the PR as summarized in Wagner *et al*. 15. Nevertheless, PR inhibition studies provided controversial results, some showing an inhibitory effect on the early phase 16, 17, 18, 19, while others did not find such effect 20, 21. HIV‐1 entry occurs in most cases by direct fusion, but the endocytotic route could make a substantial contribution to infectivity depending on the virus isolate and cell type 22, 23, 24. Although the PR has been shown to be able to process proteins at the neutral pH of the cytoplasm, as the pH optimum of the PR is acidic, the acidic pH of the lysosome is more favorable for its proteolytic action.

The capsid protein of HIV‐1 is composed of 231 amino acid residues, and consists of two independent domains: an N‐terminal core domain (residues 1–145) 25 and a C‐terminal dimerization domain (residues 151–231) 26 that are connected by a short linker region 27. The C‐terminal domain also contains the evolutionarily conserved major homology region (MHR), which is crucial for the maintenance of the structure and interactions with other viral and cellular components 26. NMR spectroscopic 25 and X‐ray crystallographic studies 26 revealed that the domain organization of the HIV‐1 CA protein is highly α‐helical. Moreover, circular dichroism spectroscopic (CD) study of a recombinant p24 protein (r‐p24) showed that under native conditions, 56% of the protein is folded into α‐helices, 3% into β‐sheets, and 20% into turns 28.

Among other viral proteins, the processed CA protein have also been found to be a substrate for the retroviral protease *in vitro*, and its processing is predicted to be dependent on a pH‐induced conformational change 12. Based on N‐terminal sequencing as well as MS identification of the fragments of recombinant CA separated by SDS/PAGE, two major CA cleavage sites were identified: Ala77/Ala78 and Leu189/Leu190 12. In addition to these sites, another study had identified additional cleavage sites: Ala22/Trp23, Gly116/Trp117, and Ala204/Leu205 14 (Fig. 1). All these cleavage sites are located outside the MHR region.

**Figure 1:**
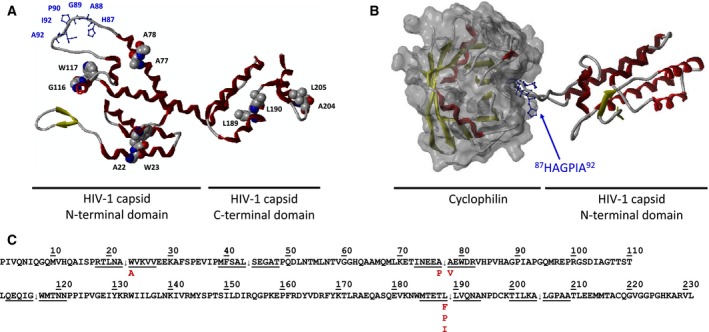
Structure of wild‐type HIV‐1 capsid protein and its complex with cyclophilin. (A) Figure shows the ribbon/tube representation of crystal structures of wild‐type HIV‐1 capsid protein (3NTE.pdb) (A) and its N‐terminal domain bound to cyclophilin A (1AK4.pdb) (B). Residues harboring the proteolytic cleavage sites are indicated by spacefill representation (residues are labeled by black color), while residues of cyclophilin A‐binding loop (^87^ HAGPIA ^92^) by ball‐and‐stick representation (residues are labeled by blue color). Color code: red, α‐helix; yellow, β‐sheet. (C) Sequence of the wild‐type CA. Identified cleavage sites are marked with arrows, and introduced amino acid changes are indicated with red letters under the wild‐type sequence.

Over the past decade, genome‐wide siRNA screenings 29, 30 and proteomic analyses 31 identified several cellular proteins which are important for HIV‐1 infection. Some of these factors such as E3 SUMO‐protein ligase (RanBP2), a nuclear pore complex protein (NUP153), transportin‐3 (TNPO3), or the cleavage and polyadenylation specificity factor subunit 6 (CPSF6) have been found to be as interaction partners of the CA protein 32, 33. One of the previously identified host‐derived cofactors, peptidyl‐prolyl isomerase cyclophilin A (CypA) has been shown to be able to bind the Gag protein of HIV‐1 and some other lentiviruses 34, 35, 36. CypA has been shown to be incorporated into HIV‐1 virions, and its incorporation was found to be essential for viral infectivity 37, 38, 39, however, further studies showed that endogenous CypA present in the target cells is more important for viral infectivity 40, 41, 42. The CypA binds to the CypA‐binding loop of the CA (^87^HAGPIA^92^ in the N‐terminal domain) (Fig. 1). The precise role of CypA in the early phase of infection is still unclear, binding of CypA to CA can be inhibited by the drug cyclosporin A (CsA) or by mutagenesis of the crucial amino acid residues of the binding regions in either one or both proteins 35, 41. Both the CsA inhibitor and mutagenesis impair the infection process in certain cell types, in a step that exists before or during reverse transcription.

The goal of the present work was based on previous specificity studies performed by us and others, to select mutations and introduce them into the major internal cleavage sites of CA, and to study how these cleavage site modifications affect the protein structure, impairing or enhancing its proteolytic susceptibility. The aim was to identify appropriate candidate mutations that can be used to clarify the potential role of CA processing at the early phase of viral infection, especially in circumstances when the virus or viral‐based vectors are utilizing the receptor‐mediated endocytotic route for infection, where the pH and the CA structure is more favorable for the proteolytic processing.

## Materials and methods

### Mutagenesis, expression, and purification of recombinant HIV‐1 capsid protein

The plasmid bearing the CA protein from HIVIIIB isolate with an N‐terminal 6‐his tag (His_6_‐HIVCA) was obtained from Dr. Carol Carter (Department of Molecular Genetics and Microbiology, S.U.N.Y. Stony Brook, USA). Mutations were introduced with the QuikChange Site‐Directed Mutagenesis Kit (Stratagene, Agilent Technologies, Santa Clara, CA, USA), using the mutagenesis primers (Integrated DNA Technologies, Coralville, IA, USA) listed in Table S1, Supporting Information. The sequences were verified by DNA sequencing performed by ABI Prism Dye Terminator Cycle Sequencing Kit and a model 3100‐Avant Genetic Analyzer (both from Applied Biosystems, Thermo Fisher Scientific Inc., Waltham, MA, USA). Protein expression in *Escherichia coli* BL21(DE3) cells (Invitrogen, Thermo Fisher Scientific Inc., Waltham, MA, USA) was induced by the addition of 1 mM IPTG) to the cultures followed by incubation at 37 °C for 3 h. Bacterial cells were suspended in lysis buffer (10 mm Tris‐HCl, 150 mm NaCl, 1 mm PMSF, pH 8.0) and then disrupted by sonication. The insoluble fraction was collected by centrifugation at 17 500 ***g*** at 4 °C for 20 min, then washed with the same buffer and centrifuged again at the same conditions. The pelleted fraction was resuspended in denaturation buffer (20 mm NaH_2_PO_4_, 500 mm NaCl, 0.1% TritonX‐100, 40 mm imidazole, 8 m urea, pH 7.5). After sonication, the solution was centrifuged at 17 500 ***g*** at 4 °C for 25 min, and then the supernatant was filtered through a 0.22‐μm pore size syringe filter (Merck, Millipore, Darmstadt, Germany) and applied to the Ni‐nitrilotriacetic acid Superflow affinity resin (Qiagen, Hilden, Germany) equilibrated in CA‐binding buffer (20 mm NaH_2_PO_4_, 500 mm NaCl, 0.1% TritonX‐100, 40 mm imidazole, pH 7.5). After consecutive, extensive washing steps using this binding buffer the recombinant protein bound to the resin was eluted with CA elution buffer (20 mm NaH_2_PO_4_, 500 mm NaCl, 500 mm imidazole, pH 7.5). The purified protein was dialyzed overnight at 4 °C against MQ water, and then concentrated with a Concentrator plus lyophilizator (Eppendorf, Hamburg, Germany). The concentrated protein was fractionated by gel filtration on Superose 12 10/300 GL column (GE Healthcare, Little Chalfont, United Kingdom) equilibrated with equilibraton buffer (50 mm NaH_2_PO_4_, 0.15 m NaCl, pH 7.0), separation was performed at a flow rate of 1 mL·min^−1^ at room temperature. Fractions with the proper purity were dialyzed and concentrated again with the same method mentioned above. The concentration of the clarified proteins were measured based on their A280 values by NanoDrop equipment (Thermo Scientific). The purity of samples was verified by SDS/PAGE using 16% polyacrylamide gels.

### Construction of pGEX‐4T‐3‐CypA expression vector

The cyclophilin A coding region was amplified from the PPIA (NM_021130) human cDNA ORF Clone (Origene, Rockville, MD, USA) using Phusion High‐Fidelity DNA polimerase (New England BioLabs, Beverly, MA, USA) with primer sets 5′‐GAGGGATCCATGGTCAACCCCACCGTGTTCTTCG‐3′ (forward) and 5′‐GCGACGCGGCCGCAATTTATTCGAGTTGTCCACAGTCAG‐3′ (reverse). The forward and reverse primers harbored a recognition sites of *Bam*HI (underlined) and *Not*I restriction endonucleases, respectively. The PCR reaction was performed as it was described before 43. After the cleavage of PCR product by *Bam*HI and *Not*I, it was ligated into pGEX‐4T‐3 plasmid (Addgene, Cambridge, MA, USA).

### Expression and purification of CypA‐GST fusion protein

The GST‐CypA coding plasmid was transformed into *E. coli* BL21(DE3) cells (Invitrogen) and the expression was induced by the addition of 0.1 mm IPTG to the cultures, and incubated at 37 °C for 3 h. After harvesting the cells by centrifugation the pellets were treated as described previously 43. The clear lysate of cells was then applied to a Bio‐Scale^TM^ Mini Profinity^TM^ GST cartridge (BIO‐RAD, Hercules, CA, USA) equilibrated in CypA‐binding buffer (10 mm NaH_2_PO_4_, 150 mm NaCl, 5 mm EDTA, pH 7.4). After consecutive, extensive washing steps with this buffer the recombinant protein bound to the resin was eluted with CypA elution buffer (20 mm glutathione, 100 mm Tris‐HCl, 5 mm EDTA, pH 8.0). The purified protein was dialyzed overnight at 4 °C, against MQ water, and then concentrated with a lyophilizator. Concentrated proteins were dissolved in MQ water again. The concentration of the clarified proteins were measured based on their A280 values by a NanoDrop equipment (Thermo Scientific). The purity of samples was verified by SDS/PAGE using 16% polyacrylamide gels.

### Expression and purification of the recombinant HIV‐1 protease

The construction of HIV‐1 PR coding region containing five stabilizing mutations (Q7K, L33I, L63I, C67A, and C95A) was described previously 44, 45. The plasmid coding for this protease was a kind gift of Dr. John M. Louis (Laboratory of Chemical Physics, NIDDK, NIH).

The HIV‐1 PR was expressed in *E. coli* BL21(DE3) cells (Invitrogen). Cells were grown at 37 °C up to an absorbance of 0.6–0.8 at 600 nm, in Luria–Bertani medium containing 100 μg·mL^−1^ ampicillin, and induced for expression with 1 mm IPTG for 3 h. Cells were harvested by centrifugation at 6000 ***g*** at 4 °C for 20 min. After removal of the supernatant, the cell pellet was treated as described previously 46. Briefly, cells were suspended in 20 volume of buffer A (50 mm Tris, 10 mm EDTA, pH 8.2), and lysed in the presence of 100 μg·mL^−1^ lysosyme by sonication on ice. The lysate was centrifuged at 20 000 ***g*** at 4 °C for 20 min, the pellet was resuspended in buffer B (buffer A containing 2 m guanidine‐HCl and 1% Triton X‐100) followed by a repeated centrifugation. Pelleted inclusion bodies were suspended in buffer A and centrifuged again. The final pellet was dissolved in buffer C (50 mm Tris, 5 mm EDTA, 7.5 m guanidine‐HCl, pH 8.0) and filtered through 0.22‐μm pore size syringe filter (Millipore). Samples were applied to a Superose 12 10/300 GL column (GE Healthcare) equilibrated in buffer D (50 mm Tris, 4 m guanidine‐HCl, 5 mm EDTA, pH 8), separation was performed at a flow rate of 0.5 mL·min^−1^ at room temperature. Peak fractions were pooled and subjected to reversed‐phase HPLC on a POROS 20 R2 column (Applied Biosystems). Purity of selected fractions was assessed by SDS/PAGE using 16% polyacrylamide gels. The protein was folded by dialysis into 0.05 m formic acid at pH 2.8 followed by a dialysis using 0.05 m sodium acetate buffer, pH 5.0. Protein concentration was measured based on its A280 value by a NanoDrop equipment (Thermo Scientific).

### Prediction of effects of mutations

Secondary structure prediction was performed using SOPMA secondary structure prediction server 47. Effects of the mutations on protein stability were predicted by the Site Directed Mutator (SDM) server 48 using the crystal structure of the wild‐type full‐length HIV‐1 capsid protein (PDB code: 3NTE) 49. Figures were prepared using Sybyl program package (Tripos Inc., St. Louis, MO, USA) run on Silicon Graphics Fuel workstations (Silicon Graphics International, Fremont, CA, USA).

### Circular dichroism spectroscopy

Circular dichroism spectra were recorded on a Jasco‐810 spectropolarimeter at room temperature in 10 mm sodium phosphate buffer (pH 7.5). CD deconvolutions were obtained using the CDSSTR analysis program 50, 51, 52, kindly provided by Dichroweb 53, 54.

### Proteolytic digestion of recombinant CA proteins by HIV‐1 protease

Recombinant CA proteins were incubated with PR at PR:CA ratio of 1:20 at pH 5.5. Reaction mixture containing recombinant His_6_‐tagged CA protein (10 μm final concentration) was incubated with recombinant HIV‐1 PR (0.5 μm final concentration) in PR reaction buffer (100 mm sodium acetate, 1 mm DTT, 1 mm EDTA, 150 mm NaCl, pH 5.5), as described previously 12. Briefly, after incubation at 37 °C for 4 h, reactions were stopped by the addition of an equal volume of 2X tricine‐SDS sample buffer containing β‐mercaptoethanol, and by heating at 95 °C for 10 min, and the samples were analyzed using 16% gradient SDS/PAGE tricine‐buffered gels (Invitrogen). In case of samples analyzed later by mass spectrometry the reactions were stopped by the addition of an equal volume of formic acid.

### Mass spectrometry

Before the measurements the samples were purified by ZipTip pipette tips loaded by C18 resin (Millipore) to remove the salt components of the HIV‐1 PR reaction buffer. The (MALDI‐TOF MS) analysis of the CA fragments was performed by a Voyager‐DETM PRO MALDI‐TOF mass spectrometer (Applied Biosystems); sinapinic acid was used as matrix. The instrument was used in the linear mode of operation.

### Limited proteolysis by trypsin

Recombinant CA proteins were incubated with trypsin at trypsin:CA ratio of 1:100 at pH 7.5 in a reaction buffer as described previously 55. Reaction mixtures contained recombinant his‐tagged CA protein (10 μm final concentration) and trypsin (100 nm final concentration). Samples were incubated at 37 °C for 2 h, reactions were stopped by the addition of an 1/6 volume of 6X SDS sample buffer containing β‐mercaptoethanol, and by heating at 95 °C for 10 min. The samples were analyzed using 16% SDS/PAGE gels.

### His6‐HIVCA pull‐down assay

After binding the His_6_‐HIVCA protein to the Ni‐nitrilotriacetic acid magnetic agarose beads (Qiagen) at 4 °C for 1 h using a PBS supplemented with 20 mm imidazole, 0.05% Tween20, pH 8.0) the mixture was incubated with the purified GST‐CypA protein in PBS at 4 °C for 1 h. The nonbound material was washed off and the bound complexes were eluted with a pull‐down elution buffer (50 mm NaH_2_PO_4_, 300 mm NaCl, 250 mm imidazole, 0.05% Tween20, pH 8.0), followed by SDS/PAGE using 14% polyacrylamide gels.

### Statistical analysis

Statistical analysis was performed using GraphPad QuickCalcs free web calculator (http://graphpad.com/quickcalcs/ttest2) (accessed January 2016).

## Results

### Rationale for the introduced mutations into the CA protein

Cleavage site mutations were based on the known specificity features of HIV‐1 PR that have been reviewed previously 56, 57. As both S1‐ and S1′‐binding cavities of HIV‐1 PR are large and hydrophobic, it was predicted that mutation of P1′Ala to Val at the Ala77/Ala78 site as well as P1 Leu to Phe at the Leu189/Leu190 cleavage site would enhance proteolysis at these sites. On the other hand, mutations of P1 residues to Pro or Ile at these sites were expected to prevent hydrolysis, as no natural or known artificial cleavage sites contain these residues at this position. Besides these mutations, we also studied the Trp23Ala mutant. The effect of this mutation has been characterized previously, showing enhanced processing of the CA protein 2; although the S1′‐binding cavity of HIV‐1 protease is relatively large, Trp appears to be too bulky to preferably fit into this site.

### Cleavage site mutant HIV‐1 capsid proteins display altered processing profiles

**Figure 2:**
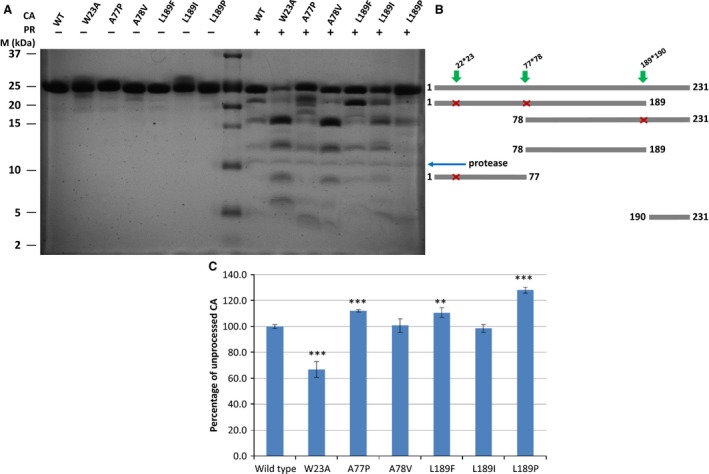
Cleavage of wild‐type and mutant HIV‐1 CA proteins by HIV‐1 PR. (A) Representative SDS/PAGE of the digested CA proteins. Recombinant CA proteins were incubated with (+) or without (−) HIV‐1 PR at 37 °C for 4 h at pH 5.5; then the reaction mixture was analyzed by SDS/PAGE. M denotes the molecular weight marker (Precision Plus Protein Dual Xtra Standard). (B) Schematic representation of proteolytic fragments produced by the proteolytic cleavage. Modified cleavage sites are marked by green arrows and missed cleavages contributing to the appearance of each fragments are represented by red crosses. Only those fragments are indicated, which have already been separated and identified previously 12. (C) Comparison of unprocessed CA amount after proteolytic digestion. Values were calculated using three independent proteolytic digestion experiments, as described in Materials and methods. Amounts of uncleaved CA after proteolytic cleavage were quantified by scanning the band densities in the gels and were calculated as percentage of total CA proteins (incubated without HIV‐1 PR). The percentage of proteolytic cleavage for the wild‐type was arbitrarily set at 100%. Error bars represent ±SD (*n* = 3). ** *P* < 0.01, *** *P* < 0.001.

To define the effects of proteolytic cleavage site mutations on the *in vitro* CA processing, we prepared mutant capsid proteins in which the P1 (A77P, L189F, L189I, and L189P mutants) or the P1′ (W23A and A78V mutants) amino acids of the cleavage sites were replaced. The mutant recombinant CA proteins were expressed in *E. coli* and subjected to proteolysis by a purified recombinant wild‐type HIV‐1 PR (Fig. 2). These cleavages were performed at pH 5.5, which is close to the lysosomal pH as well as to the pH optimum of the HIV‐1 PR.

As expected, A77P and L189P amino acid changes inhibited the proteolytic processing at these sites, as we have observed a decrease in the product band intensities or disappearance of the fragments related to these modified cleavage sites. On the other hand, the W23A mutation resulted in the accelerated proteolytic cleavage of the protein, in good agreement with previously published results 2. The modified cleavage sites became more susceptible to proteolytic cleavage in the case of A78V and L189F mutants, as indicated by the increased amount of the related fragments (78–231 and 1–189, respectively). However, the overall processing of the L189F CA was reduced, and A78V CA did not show significant alteration in its proteolytic susceptibility compared to that of the wild‐type (Fig. 2). Surprisingly, L189I mutation did not prevent processing at this site, although P1 Ile has not been found in HIV‐1 cleavage sites. This might be due to a one‐residue shift of the cleavage site to TETIL/VQNAN to produce a typical type 2 cleavage site 56, 57 for HIV‐1 PR, we, however, were unable to prove this as none of the respective peptides were recovered in MALDI‐TOF experiments (data not shown).

### *In silico* secondary structure analysis of the capsid protein

The introduced mutations could potentially alter the secondary or tertiary structural organization of the CA protein, in addition to the altered cleavage sites, the structural alterations could also be responsible for the observed changes in proteolytic processing.

Effects of mutations on the protein structure were predicted using the SDM server, which calculates a stability score analogous to the free energy difference between a wild‐type and mutant protein 48. The highest values were predicted for the A77P (−2.27 kcal·mol^−1^) and L189P (−4.85 kcal·mol^−1^) mutants, which suggested that these mutations have a highly destabilizing effect on the protein structure and may potentially cause protein malfunction.

The L189I mutation was predicted to be neutral (−0.37 kcal·mol^−1^), while A78V and L189F mutations were predicted to be slightly destabilizing (−1.57 and −0.73 kcal·mol^−1^, respectively) and cause only a slight deviation of the secondary structural organization.

Secondary structure prediction did not predict changes for W23A mutation, furthermore, SDM server predicted only slightly destabilizing effect for this mutation, as well. However, while the introduction of W23A mutation was predicted to be neutral from the viewpoint of the conformation of helix I, it was previously published that this residue is buried in the N‐terminal domain and contributes to the formation of the hydrophobic core 58. Therefore, we expected that W23A mutation would alter both the secondary and tertiary structure of CA protein, due to its critical role in stabilization of the protein structure.

### Cleavage site mutations affect the secondary structure of HIV‐1 capsid protein

**Figure 3:**
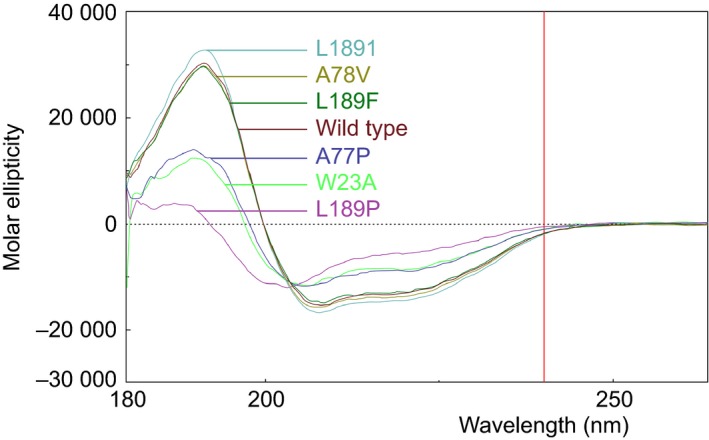
Circular dichroic spectra of recombinant HIV‐1 CA protein and its mutants in the far‐UV region at pH 7.5. The pathlength of cuvette was 0.02 cm.

To predict the effect of cleavage site mutations on the overall CA structure, we have examined the secondary structure of the proteins using CD spectroscopy. The molecular ellipticities of the spectra of the proteins were examined in the range of 180–300 nm, as indicated in Fig. 3. The spectra of mutant CA proteins were compared to that of the wild‐type CA. CD spectra were analyzed by the CDSSTR analysis program; results are summarized in Table 1. These spectra were measured at pH 7.5 in which the CA protein maintains its structural integrity 28, 59.

**Table 1:** Secondary structural organization of the wild‐type and mutant HIV‐1 capsid proteins at pH 7.5. Percentage of secondary structural elements was determined based on the results of circular dichroism spectroscopic analysis. Values are indicated based on the crystal structure of wild‐type HIV‐1 capsid protein (3NTE.pdb) and the result of secondary structure prediction (SOPMA), as well

	Crystal structure	Sec.struct. prediction	CD spectroscopy						
	Wild‐type	Wild‐type	Wild‐type	W23A	A77P	A78V	L189F	L189I	L189P
α‐helix	57	52	48	31	31	48	48	53	17
β‐strand	3	11	12	19	18	11	11	11	27
Turn	10	8	15	20	21	16	16	13	22
Unordered	30	29	25	30	30	25	25	23	34

The spectrum of the wild‐type CA protein showed positive ellipticity with a maximum at 190 nm, intense negative ellipticity with a distinct minimum at 208 nm, and a shoulder at 220 nm. The crossover point for the spectra was at 200 nm (Fig. 3). The distribution of the secondary structural elements is in good agreement with results published previously for the His_6_‐tagged CA at pH 7.5 25, 26, 28, 55, and also with the predictions made from the amino acid sequence of the wild‐type protein (Table 1). The spectra of A78V and L189F mutants showed similar features to that of the wild‐type, indicating that these proteins have the same distribution of secondary structural elements (Table 1). The spectrum of L189I mutant also exhibited similar features with a higher maximum at 191 nm, suggesting that its structure only slightly differs from that of the wild‐type protein in its α‐helix ratio (Fig. 3). The spectra of the W23A, A77P, and L189P mutants exhibited several different features compared to that of the wild‐type, such as highly reduced positive and negative ellipticity and altered crossover points of the curves, indicating the variable distribution of secondary structures (Fig. 3).

In summary, based on the results of CD, the secondary structure of the A78V, L189F, and L189I mutants show only slight differences and closely resemble that of the wild‐type (Table 1). On the other hand, W23A and A77P mutants show more pronounced changes in the percentage of secondary structural elements, the structure of L189P mutant capsid proteins highly deviated from that of the highly α‐helical wild‐type (Table 1).

Predicted effects of mutations were generally in good agreement with results obtained from CD measurements. Remarkable alterations predicted for the structures of A77P and L189P mutant capsid proteins were confirmed by CD measurements. Predicted effects of A78V, L189F, and L189I mutations corresponded well with the experimentally determined effects, and while the distribution of secondary structural elements in L189I mutant closely resembles that of the wild‐type CA, the A78V and the L189F mutants show only minor differences compared to the wild‐type protein.

### Mutant capsid proteins W23A, A77P, and L189I are more sensitive to tryptic digestion, while A78V, L189F, and L189P mutants showed similar tryptic susceptibility to that of the wild‐type CA

Previous experiments on HIV‐1 CA proteins suggested that regions exposed to tryptic digestion are crucial for protein multimerization, and altered proteolytic susceptibility to trypsin may result in the formation of CA oligomers that form structures similar to aberrant core structures *in vitro* 60.

**Figure 4:**
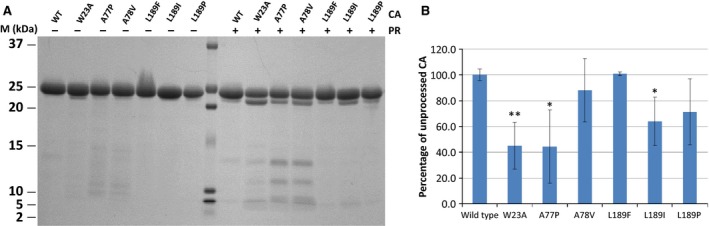
Limited tryptic digestion of the recombinant CA protein and its mutants. (A) Representative SDS/PAGE of the digested CA proteins. Recombinant CA proteins were incubated with (+) or without (−) trypsin at 37 °C for 2 h at pH 7.5; then the reaction mixture was analyzed by SDS/PAGE. M denotes the molecular weight marker (Precision Plus Protein Dual Xtra Standard). (B) Comparison of unprocessed CA amount after tryptic digestion. Values were calculated using three independent tryptic digestion experiments as described in Materials and methods. Amounts of uncleaved CA after proteolytic cleavage were quantified by scanning the band densities in the gels and were calculated as percentage of total CA proteins (incubated without HIV‐1 PR). The percentage of proteolytic cleavage for the wild‐type was arbitrarily set at 100%. The data were plotted in a bar graph. Error bars represent ±SD (*n* = 3). * *P* < 0.05, ** *P* < 0.01.

The accessibility of tryptic cleavage sites was also studied to assess structural differences between wild‐type and mutant CA proteins. Equal amount of proteins were subjected to limited proteolysis with trypsin at the same neutral pH (pH 7.5) that was used for CD spectroscopy, tryptic digestion was then followed by SDS/PAGE analysis (Fig. 4).

In case of the CA proteins bearing the A78V, L189F, or L189P mutations the efficiency of proteolytic digestion was found to be similar to that of the wild‐type, no significant difference was observed (Fig. 4). The results indicate that these mutations led to only moderate changes of the protein multimerization. However, in case of the W23A, A77P, and L189I mutants the percentage of the uncleaved CA showed significant reduction (Fig. 4). Based on these results, we can conclude that the W23A, A77P, and L189I mutant proteins are more sensitive to trypsin, compared to the wild‐type capsid protein. Therefore, it is expected that these mutant proteins could form aberrant core structures.

### Mutant HIV‐1 CA proteins with deviation in their secondary structure are still able to bind human cyclophilin A *in vitro*

**Figure 5:**
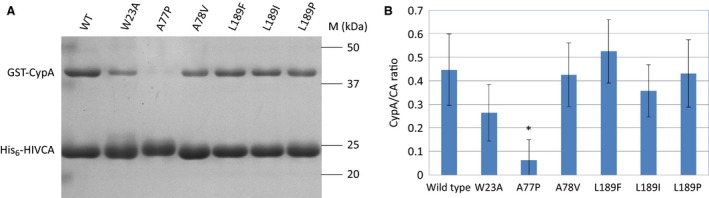
Analysis of the His_6_‐HIVCA pull‐down assay of the wild‐type and mutant HIV‐1 capsid proteins. (A) Representative SDS/PAGE of the pull‐down assays. M denotes the molecular weight marker. (B) Comparison of CypA binding ability of the HIV‐1 capsid proteins. Values were calculated using three independent His_6_‐HIVCA pull‐down assay experiments. Amount of CA and CypA proteins were calculated by scanning the band densities in the gels, and ratios of the two proteins were plotted in a bar graph. Error bars represent ±SD (*n* = 3). *P < 0.05.

The binding of CypA to the capsid protein is crucial for the proper decapsidation process in the early phase of infection. We have tested whether or not mutations of CA protein may affect the CA‐CypA interaction, and studied the binding efficiency *in vitro*. Purified His_6_‐HIVCA and GST‐CypA proteins were subjected to a His_6_‐HIVCA pull‐down assay followed by analysis of the eluted complexes by SDS/PAGE (Fig. 5A).

The amount of CypA associated with the W23A, A78V, L189F, L189I, and L189P mutants did not change significantly, while A77P mutant showed a highly reduced CypA binding ability compared to that of the wild‐type CA protein (Fig. 5B).

Based on these results, the introduced mutations (with the exception of A77P) leading to changes in the secondary structural organization (Table 1) did not affect the CypA‐binding ability of CA protein. The CypA‐binding region of CA protein is located in an exposed loop at the protein surface 26, changes of the secondary structure in a different part of the protein (Table 1, Fig. 3) may not affect the conformation or relative position of this flexible loop, and therefore, do not alter the interactions between the CypA and the binding loop of CA protein.

## Discussion

Our goal in this study was to introduce mutations into the P1 and P1′ positions of internal HIV‐1 CA protein cleavage sites to either enhance or block cleavability at a given site. Therefore, we have expressed and purified the wild‐type and modified HIV‐1 CA proteins (W23A, A77P, A78V, L189F, L189P, and L189I). Our aim was to identify appropriate candidates to be introduced into the viral genomes to study the role of CA processing in the early phase of HIV‐1 infection, or in lentivirus‐mediated gene transfers. Proteolysis at the cleavage sites containing W23A was substantially accelerated, leading to enhanced CA degradation. The A78V and L189F mutations also accelerated processing at the mutated sites, but with lack of enhanced or even decreased degradation, respectively. On the other hand, A77P and L189P mutations caused inhibition of processing at the mutated sites, resulting in decreased CA degradation.

Cleavage of the protein by HIV‐1 PR was carried out at pH 5.5, which corresponds to the pH of the endosomes 61 where the multimeric capsid will disassemble into monomers with structures altered to the ‘molten globule’ state 28, 59. This structure allows the binding of the PR to the cleavage sites which are normally hidden in the helical parts of the protein. Aside from the cleavage of the CA protein at acidic pH, it was necessary to clarify whether the mutations affect the structure of CA at a pH where it has been found to be resistant to proteolysis. Changes in the protein structure may contribute to the alteration of proteolytic processing and may even have a major effect on the stability of the core, which affects the infectivity of the virus, as well 5.

We have predicted the effects of the mutations, and besides the *in silico* analysis, we determined the secondary structural organization of the recombinant proteins by CD spectroscopy. The results of our predictions were in good agreement with results obtained from CD measurements. The A77P and L189P mutants were predicted to have a highly destabilizing effect on the protein structure, in line with the fact that proline is known to be a helix‐breaker amino acid residue. The A78V, L189F, and L189I mutations were predicted to be neutral (L189I) or slightly destabilizing. These predictions were confirmed by CD measurements: the secondary structure of A77P and L189P mutant capsid proteins showed remarkable alterations, while only slight deviations of the secondary structures were determined in case of A78V and L189F mutants, moreover, the distribution of secondary structural elements in L189I mutant closely resembled that of the wild‐type capsid protein. The only exception is the W23A mutation, which was predicted to have only a slightly destabilizing effect, CD measurement, however, detected remarkable changes in the secondary structure of this mutant protein. Trp23 has been found to be a conserved hydrophobic amino acid 2, 3, 4, so this might be the main reason behind the altered secondary structure.

Previous experiments on HIV‐1 CA proteins suggested that regions exposed to tryptic digestion are crucial for the protein multimerization, in addition, altered proteolytic susceptibility to trypsin may result in CA oligomers which form structures similar to aberrant core structures *in vitro* 55. The accessibility of trypsin cleavage sites was also used to assess structural differences in the structure of CA. We have found that the proteolytic susceptibility to trypsin did not change significantly in case of the A78V, L189F, and L189P mutants, while the W23A, A77P, and L189I mutants proved to be more prone to tryptic processing. In case of the L189F, W23A, A77P, and L189I mutants, these results are in agreement with results obtained from *in silico* analysis and CD measurements.

The importance of interaction between CA and CypA in the early phase of the replication cycle has been proved in several experiments 35, 40, 62, and observations from these experiments led to the hypothesis that CypA plays a role in the uncoating mechanism as well 63. Our results showed that CypA binding ability of W23A, A78V, L189F, L189I, and L189P mutants did not change significantly, while the A77P mutant showed a remarkable reduction in CypA binding. This was an unexpected result as A77P falls outside of the Cyp binding region.

Besides CypA, other cellular proteins like CPSF6, RanBP2, and TNPO3 have also been shown to interact with HIV‐1 CA and are able to influence virion infectivity, but these proteins do not incorporate into the virion 33. The binding site of these proteins appear to be a conserved capsid interface region formed by surface residues (N57, M66, Q67, K70, N74, and T107) of the NTD 64. While these residues are not mutated in our study, diminished interactions with the above mentioned proteins and therefore modulation of the nuclear entry of the viruses carrying the cleavage site mutations cannot be excluded.

Our results indicate that certain modifications of the main proteolytic cleavage sites of the HIV‐1 CA protein could affect not only the proteolytic susceptibility by the HIV‐1 PR but also the secondary structure, tryptic susceptibility, or CypA‐binding ability of the monomeric CA as well. While W23A, A77P, and L189P mutations can effectively modify the proteolytic susceptibility of the CA; along with the so far observed structural alterations, they are not suitable for our goal. Based on the experiments performed so far, we can conclude that the L189F mutant remains structurally unchanged; hence, it may be the best candidate for use in experiments affecting the action of HIV‐1 PR without causing structural alterations in the structure of the CA. It should be noted, that due to the multiple functions of CA, it appears to be extremely difficult to change its sequence in such a way that would influence only one feature of the protein, such as proteolytic susceptibility. Importantly, a recent study verified the extreme genetic fragility of HIV‐1 CA 65.

## Author contributions

JT, JK, and FT designed the research. FT and JK performed the experiments. FT analyzed the data with JT. JAM performed the *in silico* analysis of the secondary structure. FT wrote the paper with contributions from JT and JAM.

## Supporting information


**Table S1.** List of HIV‐1 CA mutagenesis primers.
