# Supplementary material for: Effect of internal cleavage site mutations in human immunodeficiency virus type 1 capsid protein on its structure and function
Source: FEBS Open Bio. 2016 Jun 30;6(8):847–59. doi: 10.1002/2211-5463.12094 (PMC4971840; doi:10.1002/2211-5463.12094)
Supplement: Supplementary file 1 — Table S1. List of HIV‐1 CA mutagenesis primers. [file FEB4-6-847-s001.pdf]

HIV-1 CA W23A	forward: 5’-CAC CTA GAA CTT TAA ATG CAG CGG TAA AAG TAG TAG AAG AGA AGG-3’
	reverse: 5’- CCT TCT CTT CTA CTA CTT TTA CCG CTG CAT TTA AAG TTC TAG GTG-3’
HIV-1 CA A77P	forward: 5’-GAC CAT CAA TGA GGA ACC TGC AGA ATG GGA TAG AG-3’
	reverse: 5’-CTC TAT CCC ATT CTG CAG GTT CCT CAT TGA TGG TC-3’
HIV-1 CA A78V	forward: 5’-CCA TCA ATG AGG AAG CTG TAG AAT GGG ATA GAG TAC-3’
	reverse: 5’-GTA CTC TAT CCC ATT CTA CAG CTT CCT CAT TGA TGG-3’
HIV-1 CA L189F	forward: 5’-GGA TGA CAG AAA CCT TCT TGG TCC AAA ATG CGA AC-3’
	reverse: 5’-GTT CGC ATT TTG GAC CAA GAA GGT TTC TGT CAT CC-3’
HIV-1 CA L189I	forward: 5’-AAT TGG ATG ACA GAA ACC ATC TTG GTC CAA AAT GCG AAC-3’
	reverse: 5’-GTT CGC ATT TTG GAC CAA GAT GGT TTC TGT CAT CCA ATT-3’
HIV-1 CA L189P	forward: 5’-AAT TGG ATG ACA GAA ACC CCG TTG GTC CAA AAT GCG AAC-3’
	reverse: 5’-GTT CGC ATT TTG GAC CAA CGG GGT TTC TGT CAT CCA ATT-3’
